# Differential effectiveness of residential versus outpatient aftercare for parolees from prison-based therapeutic community treatment programs

**DOI:** 10.1186/1747-597X-2-16

**Published:** 2007-05-15

**Authors:** William M Burdon, Jeff Dang, Michael L Prendergast, Nena P Messina, David Farabee

**Affiliations:** 1 University of California, Los Angeles, Integrated Substance Abuse Programs, 1640 S. Sepulveda Blvd., Suite 200, Los Angeles, California 90025, USA; 2 University of California, Los Angeles, Semel Institute for Neuroscience and Human Behavior, 760 Westwood Plaza, 47-406 NPI, Los Angeles, California 90024, USA

## Abstract

**Background:**

Research has indicated that more intense treatment is associated with better outcomes among clients who are appropriately matched to treatment intensity level based on the severity of their drug/alcohol problem. This study examined the differential effectiveness of community-based residential and outpatient treatment attended by male and female drug-involved parolees from prison-based therapeutic community substance abuse treatment programs based on the severity of their drug/alcohol problem.

**Methods:**

Subjects were 4,165 male and female parolees who received prison-based therapeutic community substance abuse treatment and who subsequently participated in *only *outpatient or *only *residential treatment following release from prison. The dependent variable of interest was return to prison within 12 months. The primary independent variables of interest were alcohol/drug problem severity (low, high) and type of aftercare (residential, outpatient). Chi-square analyses were conducted to examine the differences in 12-month RTP rates between and within the two groups of parolees (residential and outpatient parolees) based on alcohol/drug problem severity (low severity, high severity). Logistic regression analyses were performed to determine if aftercare modality (outpatient only vs. residential only) was a significant predictor of 12-month RTP rates for subjects who were classified as low severity versus those who were classified as high severity.

**Results:**

Subjects benefited equally from outpatient and residential aftercare, regardless of the severity of their drug/alcohol problem.

**Conclusion:**

As states and the federal prison system further expand prison-based treatment services, the demand and supply of aftercare treatment services will also increase. As this occurs, systems and policies governing the transitioning of individuals from prison- to community-based treatment should include a systematic and validated assessment of post-prison treatment needs and a valid and reliable means to assess the quality of community-based treatment services. They should also ensure that parolees experience a truly uninterrupted continuum of care through appropriate recognition of progress made in prison-based treatment.

## Background

Research on substance abuse treatment over the past few decades has been consistent in demonstrating that more treatment, in terms of time spent in treatment, is associated with more positive outcomes [1-4]. Data collected from three multi-program studies of drug abuse treatment in the United States (the Drug Abuse Reporting Program [DARP], 1969–1972; the Treatment Outcome Prospective Study [TOPS], 1979–1981; and the Drug Abuse Treatment Outcome Study [DATOS], 1991–1993) were consistent in showing that, overall, substance abuse treatment was effective across the major modalities of treatment (i.e., long-term residential, outpatient drug-free, outpatient methadone, and short-term inpatient), and that, generally, treatment durations of 3 months or more were associated with positive outcomes [2]. For overviews of these multi-program studies, see Simpson and Sells (DARP) [5], Hubbard et al. (TOPS) [6], and Flynn et al. (DATOS) [7].

Similar trends have been found in studies on the treatment of drug-involved offenders participating in prison-based therapeutic community (TC) treatment [8-13]. Overall, these studies found that TC participants who remained in treatment longer (up to 9 months) had lower recidivism rates, and that adding aftercare to prison-based TC treatment (i.e., increasing the length of time spent in treatment) significantly improved clients' behavior while under parole supervision and increased the chances for more successful longer-term outcomes.

Although research has been consistent in demonstrating the benefits of substance abuse treatment in terms of more time spent in treatment, it has not been able to consistently demonstrate that more intensive substance abuse treatment produces better outcomes than does less intensive treatment. Indeed, the research literature has been more consistent in demonstrating that, when staged and delivered appropriately, less intensive treatment may be just as effective as more intensive treatment.

Numerous studies that have examined differences between clients receiving treatment in different modalities have found significant improvements across modalities, while finding no significant differences between modalities on dependent measures of interest. For example, McLellan et al. [14] found that clients who attended intensive and traditional outpatient treatment programs showed significant improvements of approximately the same magnitude at 6-month follow-up. However, there were no between group differences on three of four outcome domains (reduction in alcohol and drug use, increased personal health, and reduction in public safety concerns). With respect to the fourth outcome domain (improved social function), the results were mixed. Similarly, Weinstein and Gottheil [15,16] randomly assigned patients to 3 months of Intensive Outpatient Treatment, Individual Therapy with Weekly Group, or Outpatient Individual Therapy (most intensive to least intensive). At the completion of treatment [15] and at 9-month follow-up [16], treatment retention was significantly associated with improvements on all of the outcome measures, but there were no significant differences on the dependent measures with respect to treatment modality. Hser, Evans, Huang, and Anglin [17] found that, for both residential and outpatient treatment, greater treatment service intensity and client satisfaction with treatment services were significantly associated with treatment retention and completion, which in turn was significantly related to treatment success. However, while clients in residential treatment had significantly greater service intensity, treatment retention, and treatment completion, a significantly smaller percentage of them experienced favorable outcomes compared to clients in the outpatient programs (60% vs. 73%, respectively). Similar findings have been found in studies that have varied treatment intensity within modality [18-21], with clients who received less intense forms of a treatment modality experiencing outcomes that did not significantly differ from those who received more intense forms of the same treatment modality.

Consistent with both lines of research regarding time spent in treatment and treatment intensity, Burdon at al. [8] found that among parolees from prison-based TC programs who participated in community-based treatment following release from prison (i.e., aftercare), the length of time spent in aftercare predicted 12-month return-to-prison, whereas the type of aftercare that subjects participated in (outpatient vs. residential) did not predict 12-month return-to-prison.

A consistent shortcoming of many studies showing no effect of treatment intensity or modality is that they did not examine which clients within each level of treatment intensity or modality experienced the best outcomes [22]. It may be that the overall lack of differences between varying modalities (or levels of intensity) was due to a failure to properly assess and refer clients to the appropriate modality or level of treatment intensity (i.e., treatment matching), resulting in each having a mix of clients – those who were appropriate for that particular treatment modality or level of intensity and those who were not – thus masking the true effectiveness of various modalities and intensities of treatment for specific types of clients.

In practice, when circumstances allow it to happen, matching often occurs through the referral of drug-involved individuals to substance abuse treatment programs based on the severity of their substance abuse problem and the level of need for substance abuse treatment and ancillary services [23], and there is evidence that doing so leads to increased retention in treatment and improved outcomes [24-27]. However, for the most part, clients seek or receive referrals to substance abuse treatment programs without the benefit of a systematized process that links individual needs with treatment programs or services. In many cases, clients are simply referred to the most intensive services that are available and that they will accept going to [22].

This study examined the differential effectiveness of community-based residential and outpatient treatment attended by male and female parolees who had previously participated in and paroled from prison-based therapeutic community treatment programs in the California state prison system based on the severity of their drug/alcohol problem. Consistent with previous research, which suggests that more intense treatment leads to better outcomes among clients who are appropriately matched to treatment intensity level based on the severity of their drug/alcohol problem, it was hypothesized that parolees who had a diagnosis of drug/alcohol dependence (*high severity*) would benefit more from residential aftercare than outpatient aftercare, while those who had a diagnosis of drug/alcohol abuse or no diagnosable drug/alcohol problem (*low severity*) would benefit equally from outpatient and residential aftercare. Measures of recidivism (e.g., arrests, returns to custody or prison) are often the outcome measures of most interest and relevance to policy makers, because they allow the effectiveness of treatment to also be assessed in terms of decreased costs associated with crime, arrests and adjudication, and incarceration. Accordingly, the outcome measure of interest was return-to-prison within 12 months of discharge from each client's first aftercare treatment episode.

## Methods

### Setting: treatment programs

As part of the ongoing expansion of prison-based TC treatment in California, UCLA Integrated Substance Abuse Programs (ISAP) was contracted by the California Department of Corrections (CDC; now the California Department of Corrections and Rehabilitation, CDCR) to conduct evaluations of 19 TC programs at 11 institutions. This study included data collected from all of these programs. These evaluations and the collection of all data used in this study received approval by the UCLA Institutional Review Board.

These 19 TC programs, which became operational between 1997 and 1999, provide treatment services to male and female felons at all levels of security (Level I: Minimum to Level IV: Maximum) and to male and female civil addicts. Civil addicts are inmates who are designated by the sentencing court as individuals with substance abuse problems. Compared to felon inmates, their sentences are more indeterminate in nature, and a separate parole authority makes decisions relating to their parole. Civil addicts make up only 1% of the total inmate population in California. However, because of the placement of the prison-based TC programs across institutions, they made up 37.8% of the subjects in this study.

Participation in prison-based TC treatment is mandatory for inmates who have a documented history of substance use or abuse (based on a review of inmate files) and who do not meet certain exclusionary criteria (e.g., documented in-prison gang affiliations, being housed in a Security Housing Unit within the previous 12 months for assault or weapons possession, Immigration and Naturalization Service holds). Felon inmates who parole from one of these prison-based TC programs have the option of participating in up to 6 months of community-based treatment (i.e., aftercare) at the expense of the state following their release from prison. For civil addicts, participation in aftercare is a condition of their parole. Four regionally based Substance Abuse Services Coordinating Agencies (SASCAs) under contract with CDCR act as a broker of aftercare treatment services, coordinate parolees' release and transition into aftercare, and case manage and monitor parolees during the period of time that they remain eligible for aftercare treatment services [28].

### Subjects

Subjects were 4,165 male and female parolees who received prison-based treatment in one of the 19 prison-based TC programs and who subsequently participated in *only *outpatient or *only *residential treatment following release from prison (hereinafter referred to *outpatient parolees *and *residential parolees*, respectively). Parolees who participated in various combinations of residential and outpatient treatment (n = 1,960) were excluded, thus eliminating a potential confounding factor.

### Data collection

This study incorporated data from three sources. First, client-level data were collected by the treatment providers at the time that inmates entered the prison-based TC treatment programs using an Intake Assessment (IA) instrument. The IA is designed to assess a client's pre-treatment/pre-incarceration socio-demographic background, criminality, employment, and substance use, abuse, or dependence. Adapted from the Initial Assessment developed at the Institute of Behavioral Research at Texas Christian University [29], the IA has been used extensively with criminal justice populations and provides information that is useful for both clinical and evaluation purposes. Second, aftercare participation data (i.e., admission and discharge dates, treatment modality) were collected from the SASCAs, which obtained these data from the community-based treatment providers. Third, return-to-prison data were obtained from CDCR's Offender-Based Information System (OBIS). This system contains data on each inmate's incarceration history, including prison admission dates (i.e., original admission and RTP dates), discharge dates (i.e., full discharge from correctional supervision and parole dates), and offense type (i.e., controlling conviction). All client-level data collected from treatment providers were provided to UCLA ISAP through disclosure agreements under CFR 42 Part 2, Section 2.52, which allows the treatment providers to share individually identifiable data with qualified evaluators.

### Variables

This study focused on aftercare treatment and returns-to-prison that occurred subsequent to a subject's *first *parole date following his/her *first *admission into a prison-based TC. Participation in aftercare and return-to-prison (RTP) events that followed subsequent incarcerations were not considered. This minimized potential confounding factors associated with previous in-prison residential or TC treatment and post-prison aftercare, at least to the extent that it occurred within the context of the recent expansion of in-prison TC programs in California. (Some subjects may have previously participated in prison-based substance abuse treatment programs that existed prior to the recent expansion that began in 1997. However, given the large sample size of this study, it is not expected that this occurred to an extent that would have had a significant impact on the findings of this study.)

The dependent variable of interest was *12-month RTP*, which was operationalized as the *first *return to prison (for any reason) that occurred within 12 months of a subject's discharge from his/her *first *aftercare treatment episode. This dependent variable was dichotomized and coded so that 0 = *No *and 1 = *Yes*.

The primary independent variables of interest were *alcohol/drug problem severity *and *type of aftercare*. For *alcohol/drug problem severity*, the Intake Assessment instrument contains a series of questions that allow for an unconfirmed diagnosis of alcohol abuse, alcohol dependence, drug abuse, or drug dependence. These questions were drawn directly from the DSM-IV criteria for alcohol and drug use disorders. An extracted subset of these questions make up the Texas Christian University Drug Screen instrument [30], which has demonstrated reliability and validity, and has been shown to be among the most effective instruments for identifying substance abuse and dependence disorders [30,31]. This variable was dichotomized and coded so that 0 = *Low Severity *(Abuse/None) and 1 = *High Severity *(Dependence).

*Type of aftercare *was also dichotomized and coded so that 0 = *Residential Only *and 1 = *Outpatient Only*. Participation in aftercare was operationalized as any documented admission to a community-based treatment program subsequent to a subject's *first *parole date following his/her *first *admission into a prison-based TC and prior to any RTP event.

Other independent variables of interest included demographic background, criminal background, drug use, and time spent in drug treatment (i.e., prison-based treatment and aftercare). *Mental disorder *reflected a CDCR classification that is assigned to inmates who are assessed as having a co-occurring mental disorder that can be controlled through therapy and/or medications. *County of parole *classified the counties to which inmates paroled as urban, suburban, or rural based on the percentage of the population in each county that lived in unincorporated areas [32] (0–25%, urban; 26–50%, suburban; more than 50%, rural).

### Data analyses

Using a standardized process detailed in Tabachnick and Fidell [33], all relevant variables were examined prior to analyses for accuracy and to identify potential outliers that could impact the subsequent statistical analyses.

Comparative descriptive statistics were computed between residential parolees and outpatient parolees using independent samples t-tests and chi-square analyses. Differences between the two groups were computed on variables chosen for the multivariate logistic regression analyses described below. Chi-square analyses were conducted to examine differences in 12-month RTP rates between and within the two groups of parolees (residential parolees and outpatient parolees) based on alcohol/drug problem severity (low severity, high severity). Cases with missing data (N = 214) were excluded. Finally, logistic regression analyses were performed to determine if aftercare modality (outpatient only vs. residential only) was a significant predictor of 12-month RTP rates for subjects who were classified as low severity versus those who were classified as high severity.

This study involved a complex design in which the subjects were nested within 19 different treatment programs. The average cluster size across these 19 programs was 208. When data are clustered in this manner, there are two sources of variance that need to be considered: variability of participants within a cluster (program) and variability between clusters (programs). Together, these sources of variance can increase standard errors, confidence intervals and *p*-values. If the clustered design is not taken into account in the analyses, the assumption of independent observations is violated, which can lead to a greater risk of false positives or Type I errors. To address this issue, two separate logistic regression analyses were performed using Stata Intercooled Version 9.0 in which robust standard errors were calculated to account for the clustered design and the resulting variability among participants within and between the 19 different treatment programs. The first analysis included only subjects who were classified as low severity; the second analysis included only subjects who were classified as high severity.

## Results

### Descriptive statistics

Given the large sample size, even small differences between outpatient and residential parolees that did not appear practically relevant achieved statistical significance. Therefore, only those differences that attained both practical and statistical significance are summarized below and in Table 1.

**Table 1:** Comparative Descriptive Statistics: Outpatient vs. Residential

	**Outpatient**	**Residential**	**Tests of Significance**
			
**N**	1,342	2,823	
**Background**			
**Age (Mean/SD)**	37.9 (8.4)	36.0 (8.4)	*t*[4,163] = 6.630, *p *< .001
**Sex**			
**Male**	68.3%	59.5%	*X*^2^[1, 4,165] = 29.395, *p *< .001
**Female**	31.7%	40.5%	
			
	100.0%	100.0%	
			
**Criminal Background**			
**Lifetime years in prison (Mean/SD)**			
2 years or less	36.1%	46.2%	*X*^2^[5, 3,897] = 41.301, *p *< .001
2.01 to 4.00 years	19.9%	18.8%	
4.01 to 6.00 years	16.1%	12.6%	
6.01 to 8.00 years	8.4%	6.1%	
8.01 to 10.00 years	6.1%	5.8%	
More than 10 years	13.4%	10.5%	
			
	100.0%	100.0%	
			
Most recent offense*			
Violent	15.9%	9.9%	*X*^2^[3, 4,162] = 43.810, *p *< .001
Property	28.8%	29.8%	
Drug	48.7%	55.7%	
Other	6.6%	4.6%	
			
	100.0%	100.0%	
			
**Next Arrest Strike Number**			
First Strike	24.2%	18.2%	*X*^2^[4, 3,967] = 36.326, *p *< .001
Second Strike	11.3%	10.0%	
Third Strike	11.5%	9.0%	
None of the Above	35.8%	41.5%	
Don't Know	17.2%	21.3%	
			
	100.0%	100.0%	
			
**Level of Security**			
Felons	87.3%	50.3%	*X*^2^[1, 4,165] = 531.586, *p *< .001
Civil Addicts	12.7%	49.7%	
			
	100.0%	100.0%	
			
**Drug Use**			
**Alcohol/drug disoder**			
Neither/Abuse	23.0%	14.5%	*X*^2^[1, 3,951] = 44.166, *p *< .001
Dependence	77.0%	85.5%	
			
	100.0%	100.0%	
			
**Most frequently cited problem**			
**drug prior to incarceration**			
Alcohol	10.0%	7.7%	*X*^2^[4, 3,933] = 56.176, *p *< .001
Cocaine/Crack	23.4%	25.1%	
Meth/Amphetamine/Stimulants	29.8%	38.6%	
Opiates	18.6%	17.4%	
Other drug**	18.2%	11.2%	
			
	100.0%	100.0%	
			
**Frequent user prior to prison**	77.6%	84.0%	*X*^2^[1, 4,165] = 25.296, *p *< .001
**Involvement in Drug Treatment**			
**Time in prison treatment**			
0 to 90 days	7.1%	9.1%	*X*^2^[3, 3,520] = 30.597, *p *< .001
91 to 180 days	18.1%	13.3%	
181 to 270 days	29.8%	25.1%	
More than 270 days	45.0%	52.5%	
			
	100.0%	100.0%	
			
**Time in Aftercare: Episode 1**			
0 to 90 days	58.0%	67.0%	*X*^2^[1, 4,165] = 31.836, *p *< .001
More than 90 days	42.0%	33.0%	
			
	100.0%	100.0%	
			
**Attended Aftercare: Post-Episode 1**	16.2%	21.3%	*X*^2^[1, 4,165] = 15.301, *p *< .001

* Data obtained from CDC Offender-Based Information System.

** Includes tranquilizers, hallucinogens, other drugs

Outpatient parolees were on average older compared to residential parolees and more often male. With respect to criminal background, outpatient parolees were more likely to be felons who committed a violent or other offense and whose next felony conviction would represent a felony "strike," which would likely result in a longer period of incarceration. Residential parolees were more likely to be civil addicts who committed a drug-related or property offense, and who were less likely to be facing a felony strike with their next conviction. Largely because of this, outpatient parolees had significantly more self-reported lifetime years in prison than did residential parolees. A significantly larger percentage of the outpatient parolees were paroled to urban counties, compared to the residential parolees, who were paroled to suburban or rural counties.

With respect to drug use, outpatient parolees were significantly more likely to have no drug/alcohol problem or to be drug/alcohol abusers (i.e., low severity). They were also significantly more likely to report alcohol, opiates, or other drugs as their problem drug. Residential parolees were significantly more likely to be drug/alcohol dependent (i.e., high severity), and significantly more likely to report methamphetamine or cocaine/crack as their problem drug. Compared to outpatient parolees, residential parolees were also significantly more likely to have been using drugs frequently prior their incarceration (i.e., at least 1–5 times per week).

Finally, with respect to involvement in drug treatment, residential parolees were significantly more likely to have spent more than 270 days (9 months) in prison-based treatment. Outpatient parolees were significantly more likely to have spent more than 90 days in their first aftercare treatment episode, although residential parolees were significantly more likely to have participated in additional aftercare treatment following the completion of their first aftercare treatment episode.

### Inferential statistics

Table 2 contains the results of chi-square analyses that were conducted to examine differences in 12-month RTP rates between and within the two groups of parolees (outpatient parolees and residential parolees) based on alcohol/drug problem severity (low severity, high severity). The overall 12-month RTP rate for all subjects was 40.5%. Within problem severity category, there were no significant differences in 12-month RTP rates between outpatient parolees and residential parolees. Similarly, within each group of parolees, there were no significant differences in 12-month RTP rates based on alcohol/drug problem severity.

**Table 2:** 12-Month RTP Rates by Aftercare Type and Alcohol/Drug Problem Severity

	**Outpatient**	**Residential**		**Chi-square**	
	**Only**	**Only**	**Total**	**p-value**	
**Low Severity**					
N	287	391	678		
%	40.1%	38.1%	38.9%	*p *= .605	
**High Severity**					
N	959	2,314	3,273		
%	40.6%	41.0%	40.9%	*p *= .812	
**Total**					
N	1,246	2,705	3,951		
%	40.4%	40.6%	40.5%	*p *= .933	
**Chi-square p-value**	*p *= .881	*p *= .279	*p *= .349		

The results of the logistic regression analyses appear in Table 3. For subjects classified as low severity, *Age, Sex, Living with prior to prison, Mental disorder, Lifetime years in prison, Time in prison treatment*, and *Time in aftercare: episode 1 *emerged as significant predictors of 12-month RTP. For each additional year in age, the odds of being returned to prison within 12 months decreased by 6%. Females were 59% less likely than males to have been returned to prison within 12 months. Compared to subjects who lived alone prior to incarceration, those who lived with their friends were 46% less likely to have been returned to prison within 12 months. Subjects with a mental disorder were 67% more likely to be returned to prison within 12 months than those without a mental disorder. Compared to subjects who had spent a total of 0–2 lifetime years in prison, those who had spent 4.01–6.0, 8.01–10.0, and greater than 10 lifetime years in prison were more likely to have been returned to prison within 12 months (125%, 234%, and 183%, respectively). Compared to subjects who had spent 0–90 days in prison treatment, the odds of being returned to prison within 12 months was 56% lower for those who had spent 181–270 days in prison treatment. Finally, the odds of being returned to prison within 12 months was 60% higher for subjects who spent more than 90 days in their first aftercare treatment episode than for those who spent less than 90 days. *Aftercare: post episode 1 *and *Type of aftercare *were not significant predictors of 12-month RTP.

**Table 3:** Logistic Regressions

	**Low Severity**		**High Severity**	
		
**Variables**	**OR**	**95% CI**	**OR**	**95% CI**
		
**Age**	0.94*	**(0.92, 0.96)**	0.96*	**(0.95, 0.97)**
**Years of Education**	1.04	(0.92, 1.19)	0.97	(0.92, 1.01)
**Sex**				
Male	1	(-)	1	(-)
Female	0.41*	**(0.23, 0.73)**	0.6*	**(0.49, 0.72)**
**Ethnicity**				
Black	1	(-)	1	(-)
Hispanic	0.85	(0.48, 1.52)	1.03	(0.84, 1.27)
White	0.77	(0.46, 1.29)	0.94	(0.78, 1.13)
Other	0.39	(0.07, 2.01)	0.55*	**(0.35, 0.88)**
**Marital Sstatus**				
Never Married	1	(-)	1	(-)
Married/Living as married	1.12	(0.58, 2.15)	1.01	(0.83, 1.24)
Previously Married/Separated	1.15	(0.66, 2.02)	1.08	(0.83, 1.41)
**Living with Prior to Prison**				
Alone	1	(-)	1	(-)
Family/Relatives	1.24	(0.90, 1.69)	1.08	(0.81, 1.44)
Friends	0.54*	**(0.30, 0.96)**	1.30*	**(1.02, 1.65)**
Other	1.09	(0.55, 2.18)	1.40	(0.97, 2.04)
**Housing prior to prison**				
Homeless	1	(-)	1	(-)
Boarding House/Hotel	0.98	(0.33, 2.95)	1.17	(0.84, 1.63)
House/Apartment/Condo	0.48	(0.21, 1.10)	0.81	(0.58, 1.13)
Other	0.58	(0.20, 1.71)	1.24	(0.91, 1.70)
**Employed Before Incarceration**				
No	1	(-)	1	(-)
Yes	0.87	(0.53, 1.43)	0.91	(0.75, 1.12)
**Mental Disorder**				
No	1	(-)	1	(-)
Yes	1.67*	**(1.07, 2.62)**	1.26*	**(1.04, 1.52)**
**Lifetime Years in Prison**				
0.00–2.00	1	(-)	1	(-)
2.01–4.00	1.49	(0.92, 2.43)	1.09	(0.85, 1.41)
4.01–6.00	2.25*	**(1.29, 3.94)**	1.38*	**(1.04, 1.83)**
6.01–8.00	1.75	(0.66, 4.66)	1.64*	**(1.28, 2.10)**
8.01–10.00	3.34*	**(1.71, 6.55)**	1.43*	**(1.10, 1.86)**
10.01+	2.83*	**(1.36, 5.88)**	1.82*	**(1.43, 2.31)**
**Most Recent Offense**				
Violent	1	(-)	1	(-)
Property	0.89	(0.35, 2.27)	0.91	(0.65, 1.28)
Drug	1.07	(0.48, 2.37)	0.90	(0.63, 1.29)
Other	0.79	(0.20, 3.15)	0.75	(0.46, 1.22)
**Next Arrest Strike Number**				
First Strike	1	(-)	1	(-)
Second Strike	0.85	(0.33, 2.21)	1.16	(0.82, 1.63)
Third Strike	0.71	(0.36, 1.38)	0.91	(0.66, 1.27)
None	0.59	(0.33, 1.06)	1.08	(0.84, 1.39)
I Don't Know	0.66	(0.26, 1.63)	0.94	(0.67, 1.32)
**Level of Security**				
Felons	1	(-)	1	(-)
Civil Addicts	0.89	(0.57, 1.39)	0.60*	**(0.47, 0.76)**
**Primary Problem Drug**				
Alcohol	1	(-)	1	(-)
Cocaine	0.88	(0.33, 2.40)	1.05	(0.68, 1.62)
Meth	0.70	(0.26, 1.87)	0.72	(0.46, 1.13)
Opiates	0.91	(0.38, 2.21)	0.88	(0.54, 1.44)
Other	0.55	(0.21, 1.41)	0.92	(0.55, 1.52)
**Frequent User Prior to Prison**				
No	1	(-)	1	(-)
Yes	1.34	(0.82, 2.18)	1.12	(0.87, 1.45)
**Time in Prison Treatment (days)**				
0–90	1	(-)	1	(-)
91–180	1.28	(0.65, 2.50)	0.82*	**(0.68, 0.98)**
181–270	0.44*	**(0.20, 0.94)**	0.60*	**(0.46, 0.78)**
270+	0.53	(0.21, 1.36)	0.60*	**(0.46, 0.80)**
**Time if Aftercare: Episode 1 (days)**				
0–90	1	(-)	1	(-)
91+	0.40*	**(0.21, 0.76)**	0.40*	**(0.32, 0.49)**
**Aftercare: Post-Episode 1 (days)**				
None	1	(-)	1	(-)
1 or more	0.75	(0.42, 1.34)	0.64*	**(0.53, 0.79)**
**Type of Aftercare**				
Residential	1	(-)	1	(-)
Outpatient	1.09	(0.63, 1.87)	0.84	(0.66, 1.07)

* p < .05.

For subjects classified as high severity, *Age, Sex, Ethnicity, Living with prior to prison, Mental disorder, Lifetime years in prison, Level of security, Time spent in prison treatment, Time in aftercare: episode 1*, and *Aftercare: post-episode 1 *emerged as significant predictors of 12-month RTP. For each additional year in age, the odds of being returned to prison within 12 months decreased by 4%. Females were 60% less likely than males to have been returned to prison within 12 months. Compared to subjects who lived alone prior to incarceration, the odds of being returned to prison within 12 months was 30% higher among those who lived with friends. Subjects with a mental disorder were 26% more likely to have been returned to prison within 12 months compared to those without a mental disorder. Compared to subjects who had spent a total of 0–2 lifetime years in prison, those who had spent 4.01–6.0, 6.01–8.0, 8.01–10.0, and more than 10 lifetime years in prison were more likely to have been returned to prison within 12 months (38%, 64%, 43%, and 82%, respectively). Civil addicts were 40% less likely than felon offenders to have been returned to prison within 12 months. Subjects who had spent 91–180, 181–270, and more than 270 days in prison treatment were significantly more likely to have been returned to prison within 12 months than those who spent 90 days or less in prison treatment (18%, 40%, and 40%, respectively). The odds of being returned to prison within 12 months was 60% higher for subjects who spent more than 90 days in their first aftercare treatment episode than for those who spent less than 90 days. Finally, those who participated in additional aftercare following the first aftercare treatment episode were 36% less likely to have been returned to prison within 12 months than those who did not. *Type of aftercare *was not a significant predictor of 12-month RTP.

## Discussion

One of the hypotheses was confirmed, one was not. Subjects classified as low severity (i.e., had no diagnosable drug/alcohol problem or a drug/alcohol abuse problem) benefited equally from outpatient and residential aftercare. However, contrary to what was expected, subjects classified as high severity (i.e., were drug/alcohol dependent) also benefited equally from outpatient and residential aftercare.

The results of the chi-square analyses (Table 2) showed no significant differences in the 12-month RTP rates among parolees who attended only outpatient treatment and those who attended only residential treatment, regardless of the severity of their drug/alcohol problem. The logistic regression analyses further showed that, after controlling for static demographic and criminal background variables, drug use behaviors, time spent in prison-based treatment, and time spent in aftercare (episode 1 and post-episode 1), the type of aftercare that subjects participated in (i.e., only outpatient versus only residential treatment) was not a significant predictor of 12-month RTP rates.

In both regression analyses, time spent in treatment (prison treatment and aftercare) emerged as a significant predictor of 12-month RTP. These results are consistent with previous research that has highlighted the importance of participation and retention in aftercare in combination with prison-based treatment as a means of ensuring successful treatment outcomes as measured by RTP [8-11,13,34-36].

Most importantly, as it relates to the hypotheses of this study, the results of the analyses yielded no evidence of differential effectiveness between outpatient and residential aftercare in reducing recidivism among drug-involved offenders following their release from prison-based treatment, regardless of level of drug/alcohol problem severity. Within a 95% confidence interval, the odds of being returned to prison within 12 months was not appreciably different from 1.00 for those who participated in only outpatient aftercare compared to those who participated in only residential aftercare. However, other factors that characterize the treatment initiative in California, and that were not or could not be accounted or controlled for in this study, need to be considered before embracing the conclusion that there is no differential effectiveness between residential and outpatient aftercare treatment.

Most research that has demonstrated the effectiveness of prison-based treatment followed by aftercare has focused on situations where there were only one or very few in-prison treatment programs or providers and only one or very few aftercare treatment programs [9-13]. Such scenarios are likely to be characterized by a high level of coordination between the in-prison and aftercare treatment providers. In addition, parolees entering aftercare are more likely to experience continuity of treatment, with aftercare treatment services picking up where prison-based treatment stopped, and are more likely to go through the in-prison and post-prison treatment experience in cohorts. Also, due to the limited number of aftercare programs/providers, the variability in the quality of the treatment services received by parolees who attend aftercare can be attenuated.

Such scenarios contrast to the situation in California, where the rapid expansion of prison-based TC treatment since 1997 has resulted in a similar rapid growth in the number of community-based programs providing treatment services to individuals paroling from the prison-based TC programs. In this study, 455 different community-based treatment programs (164 outpatient and 291 residential) delivered treatment services to the 4,165 parolees included in the analyses, who paroled from just 19 different prison-based TC programs. This computes to an average of 9.2 parolees per community-based program.

The "continuum of care" construct rests on the notion that the transition of parolees from prison-based treatment to community-based treatment be "seamless" (i.e., uninterrupted) [37]. However, anecdotal data collected as part of the process evaluations conducted on California's prison-based treatment initiative (1997–2004) suggests that parolees entering community-based treatment programs often felt as though they were not given credit for the "uninterrupted" progress that they made in prison-based treatment, that treatment in the community did not pick up where treatment in prison left off. To the extent that this occurred, it may have contributed to increased client dissatisfaction with aftercare treatment and increased dropout rates, triggering a perception of failure on behalf of the parolee (who voluntarily entered treatment) and possibly leading to a return to criminal activity, drug use, and ultimately reincarceration.

The large number of community-based treatment programs also raises the question of variability in the quality of aftercare treatment services provided to parolees by community-based providers – a measure that is difficult to capture. In California, residential programs are required to be licensed, while non-residential (i.e., outpatient) treatment programs are not. However, neither residential nor non-residential programs are required to be certified by the state [38], which would ensure that a program is delivering a minimal level of service quality. While CDCR and the four regionally-based SASCAs do take into consideration the quality of treatment services provided by individual community-based treatment programs when making referrals and placement decisions, the current system for transitioning parolees from prison-based treatment programs to the large network of community-based treatment programs was not designed to assess the quality of aftercare treatment services, and it does not ensure that community-based treatment programs take into account progress that parolees made in prison-based treatment.

Finally, the process of transitioning parolees from prison- to community-based treatment in California also does not include a formal systematic assessment process for matching parolees' needs with community-based treatment programs or services. Subjects in this study consisted of parolees who attended *only *residential or *only *outpatient aftercare. The majority of these subjects (68%) attended only residential aftercare. There were an additional 1,960 parolees who attended a combination of both residential and outpatient aftercare following their release from prison and who were not included in this study. Residential treatment constituted the first aftercare treatment episode for 92% of these 1,960 parolees. Combined, these facts indicate that, following conventional wisdom, parolees were most often referred to and encouraged to attend residential treatment in the community (i.e., the more intensive treatment modality).

There currently exist no validated assessments that are designed specifically for substance-abusing parolees who are encouraged or required to participate in treatment after they are released from prison. However, recently, as part of the ongoing NIDA-funded Criminal Justice – Drug Abuse Treatment Studies (CJ-DATS) research initiative, researchers at several sites across the country are testing the efficacy of the *Inmate Pre-Release Assessment *(IPASS) [39] to match paroling offenders to an appropriate modality of aftercare. Specifically designed as a pre-release risk measure for prison-based substance abuse treatment graduates, the IPASS takes into account inmates' historical drug use and criminal activity, as well as performance in prison-based treatment. If successful, the IPASS instrument will constitute the first validated tool for assessing treatment needs of substance abusing parolees being released from prison and for guiding referrals to effective aftercare treatment.

With respect to the limitations of this study, the measure of alcohol/drug problem severity, as well as some measures relating to demographic background and criminal background, were based on self-report. Although questions are often raised about self-report data, prior research indicates that self-report interviews, when properly conducted, are generally reliable and valid in measuring drug and alcohol use [40-42] and criminal involvement [43-45]. In addition, some self-report measures were compared to official records. Where this occurred, analyses comparing self-report data to those obtained from the official records showed no differences (e.g., type of offense). Combined, these factors alleviated concerns regarding the veracity of self-report data.

This study was concerned with assessing the differential effectiveness of residential versus outpatient aftercare treatment among those subjects who actually participated in aftercare treatment. Thus, the potential impact of selection bias on the results is limited to factors that may have influenced the decision to participate in residential or outpatient aftercare, but not the decision to participate or not participate in aftercare. Despite the emphasis on referring parolees to residential treatment, most parolees do play a role in choosing the modality of aftercare treatment that they participate in (i.e., residential or outpatient). With respect to selection bias and its impact on the choice of treatment modality, the logistic regression analyses controlled for a full array of variables that likely influence the decision regarding which modality of treatment to participate in. However, there may be other unknown individual- and system-level factors that were not measured in this study, and thus not controlled for, that influence the decision to participate in residential or outpatient aftercare treatment.

Finally, although the logistic regression analyses accounted for the clustered design and the resulting variability among participants within and between the different in-prison treatment programs, relevant program-level variables were not collected and thus were outside the scope of this study. Future studies should examine various programmatic differences using multilevel modeling techniques to explain the variation between different in-prison treatment programs (e.g., quality of the individual treatment program).

## Conclusion

The purpose of this study was to examine the differential effectiveness of community-based residential and outpatient treatment attended by male and female parolees who had previously participated in and paroled from prison-based therapeutic community treatment programs. The results of this study highlight the need for empirical research that examines more closely the "continuum of care" construct, including the assumptions that underlie it and the systems, policies, and procedures that are employed to ensure the transition from prison- to community-based treatment is as continuous and seamless as possible. To the extent that various states and the federal prison system further expand prison-based treatment services, the demand for and supply of aftercare treatment services will also increase, resulting in scenarios where (similar to the current scenario in California) large numbers of paroles are transitioning from a relatively small number of prison-based treatment programs to much larger number of community-based treatment programs that vary widely in terms of modality, intensity, and quality of treatment services. As this occurs, policy- and decision-makers will need to implement policies that ensure the process of transitioning individuals from prison- to community-based treatment includes a systematic and validated assessment of post-prison treatment needs, which in turn should guide the referral process. In addition, as the number of community-based treatment programs increases to meet the treatment demands of larger numbers of parolees from prisons who are in need of substance abuse treatment, policies need to be implemented that ensure the valid and reliable assessment of the quality of community-based treatment services and that ensure that parolees experience a truly *uninterrupted *continuum of care through appropriate recognition of progress made in prison-based treatment.

## Competing interests

The author(s) declare that they have no competing interests.

## Authors' contributions

WMB designed this study, oversaw data analyses, and wrote the manuscript. JD designed and performed the statistical analyses, wrote relevant portions of the methods and results sections of the manuscript, and provided editorial feedback on other parts of the manuscript. MLP and NPM provided feedback on data analyses and editorial assistance in the preparation of the manuscript. WMB, NPM, and DF served as Project Directors for the evaluation studies of the 19 prison-based TC programs from which the data were drawn for this study. MLP served as Principal Investigator for these evaluation studies. All authors have read and approved this manuscript.
